# Study of the Active Components and Molecular Mechanism of *Tripterygium wilfordii* in the Treatment of Diabetic Nephropathy

**DOI:** 10.3389/fmolb.2021.664416

**Published:** 2021-06-07

**Authors:** Lin Wang, Zheyi Wang, Zhihua Yang, Kang Yang, Hongtao Yang

**Affiliations:** ^1^ Graduate School, First Affiliated Hospital of Tianjin University of Traditional Chinese Medicine, Tianjin, China; ^2^ Dongzhimen Hospital, Beijing University of Chinese Medicine, Beijing, China

**Keywords:** *Tripterygium wilfordii*, diabetic nephropathy, active ingredients, molecular mechanism of action, network pharmacology

## Abstract

We aimed to explore the active ingredients and molecular mechanism of *Tripterygium wilfordii* (TW) in the treatment of diabetic nephropathy (DN) through network pharmacology and molecular biology. First, the active ingredients and potential targets of TW were obtained through the Traditional Chinese Medicine Systems Pharmacology Database and Analysis Platform (TCMSP) and related literature materials, and Cytoscape 3.7.2 software was used to construct the active ingredient-target network diagram of TW. Second, the target set of DN was obtained through the disease database, and the potential targets of TW in the treatment of DN were screened through a Venn diagram. A protein interaction network diagram (PPI) was constructed with the help of the String platform and Cytoscape 3.7.2. Third, the ClueGO plug-in tool was used to enrich the GO biological process and the KEGG metabolic pathway. Finally, molecular docking experiments and cell pathway analyses were performed. As a result, a total of 52 active ingredients of TW were screened, and 141 predicted targets and 49 target genes related to DN were identified. The biological process of GO is mediated mainly through the regulation of oxygen metabolism, endothelial cell proliferation, acute inflammation, apoptotic signal transduction pathway, fibroblast proliferation, positive regulation of cyclase activity, adipocyte differentiation and other biological processes. KEGG enrichment analysis showed that the main pathways involved were AGE-RAGE, vascular endothelial growth factor, HIF-1, IL-17, relaxin signalling pathway, TNF, Fc epsilon RI, insulin resistance and other signaling pathways. It can be concluded that TW may treat DN by reducing inflammation, reducing antioxidative stress, regulating immunity, improving vascular disease, reducing insulin resistance, delaying renal fibrosis, repairing podocytes, and reducing cell apoptosis, among others, with multicomponent, multitarget and multisystem characteristics.

## Introduction

According to data surveys, it is inferred that by 2030, diabetes will become the seventh most common cause of death in the world (Rao et al., 2019), and diabetic nephropathy (DN) is one of its most serious complications and is the main cause of end-stage renal disease (Liu et al., 2009; Toth-Manikowski and Atta, 2015). On average, there is one DN patient for every three diabetic patients, and more than 30% of DN patients require kidney dialysis or kidney transplantation, which will place a huge economic burden on individuals and society (Montero et al., 2016). DN mainly manifests as proteinuria, a decreased glomerular filtration rate, and nephrotic syndrome (Montero et al., 2016). Currently, the recommended treatments for DN are to control blood pressure and blood sugar, mainly by administering renin angiotensin aldosterone system (RAAS) inhibitors, sodium glucose cotransporter 2 (SGLT2) inhibitors, and glucagon-like peptide 1 (GLP1) receptor agonists. Although these strategies have shown encouraging results in DN, there are still many diabetic patients who continue to progress towards end-stage kidney disease (ESKD) (Kato and Natarajan, 2019; Barrera-Chimal and Jaisser, 2020). Moreover, due to the complex environment of diabetes, no single therapy can cure DN. Multiple interventions should be used to jointly intervene in the pathological process. Therefore, new strategies are needed to supplement existing interventions (Batu Demir and Cooper, 2016).

Traditional Chinese medicine (TCM) also provides an effective treatment for DN. In China, Chinese herbal medicine is widely used in the treatment of DN, among which *Tripterygium wilfordii* (TW) is the most commonly used. TW has been used in TCM for more than two thousand years for the treatment of rheumatoid arthritis, autoimmune diseases and kidney diseases (Chen, 2001; Luo et al., 2019). Modern pharmacological studies have shown that TW and its extracts have anti-inflammatory and immunosuppressive effects (Ma et al., 2007; Ziaei and Halaby, 2016; Chen et al., 2018). It can effectively protect the kidneys and reduce urine protein and podocyte damage. It is potentially effective and safe drug for the treatment of DN patients (Liu, 2009; Ge et al., 2013). However, the mechanism of TW in the treatment of DN has not been fully elucidated. This article explores the mechanism of TW in the treatment of DN based on network pharmacology, aiming to provide a reference for clinical applications and basic research.

## Materials and Methods

### Screening of Active Components and Targets of TW and Construction of the Network

All of the chemical constituents of TW were searched in the Traditional Chinese Medicine Systems Pharmacology Database and Analysis Platform (TCMSP). The TCMSP database is the most commonly used database for the retrieval of Chinese medicine ingredients and it describes the relationships between drugs, targets and diseases (Ru et al., 2014; Zhu et al., 2018). This database includes 500TCMs from the 2010 edition of the pharmacopoeia and 3,069 compounds (Huang et al., 2017). The active components of TW were screened according to the (ADME) parameters of "oral bioavailability (OB) ≥ 30%, drug-like (DL) ≥ 0.18”, and the action targets of the active components were predicted. Combined with related research, these results should be supplemented. The predicted targets were further standardized through the UniProt database and corrected to the official gene names (Jin et al., 2018). Cytoscape 3.7.2 software was used to construct a network diagram of TW active ingredient targets, and the key compounds were screened according to their topological parameters.

### DN-Related Gene Screening

The DN-related target proteins were collected from the following four widely recognized disease databases: 1) Therapeutic Target Database (TTD) (http://db.idrblab.net/ttd/) (Chen et al., 2002), 2) DrugBank (https://www.drugbank.ca/) (Wishart et al., 2008), 3) DisGeNET (https://www.disgenet.org/) (Piñero et al., 2016), and 4) the National Center for Biotechnology Information (NCBI) (https://www.ncbi.nlm.nih.gov/) (Benson et al., 1990). We searched the four databases with the keyword “diabetic nephropathy” and set the species to “*Homo sapiens*”.

### Prediction of Potential Targets of TW in the Treatment of DN

Venn diagrams (http://bioinfogp.cnb.csic.es/tools/venny/index.html) are commonly used to display list comparisons (Oliveros, 2007). They are widely used in biology to illustrate the differences between gene lists originating from different differential analyses (Bardou et al., 2014). Through the Venn diagram, the obtained targets of TW and the targets of DN intersected, and the intersectional targets were considered potential therapeutic targets of TW in DN.

### PPI Network Construction and Core Target Screening

The common target genes obtained from the Venn graph were imported into the STRING database, which is a database for predicting protein–protein interactions, and the species were selected as "*Homo sapiens*" to obtain the interaction relationship between the targets. The confidence level adopts the system default "score > 0.4″, saves it in TSV format, and imports Cytoscape 3.7.2 software to build a more advanced protein interaction network diagram. A network analyser is used to calculate topology parameters, such as the node degree value, to filter the core targets.

### Gene Ontology and Kyoto Encyclopedia of Genes and Genomes (KEGG) Enrichment Analysis (Gene Function and Pathway Enrichment Analysis)

Gene Ontology (GO) is an international standard system used to classify gene functions. It divides gene functions into three aspects: molecular function (MF), cell composition (CC) and biological process (BP) (Ashburner et al., 2000). Kyoto Encyclopedia of Genes and Genomes (KEGG) is a set of artificially drawn pathway maps representing molecular interactions and reaction networks.

To predict the molecular mechanism of TW in the treatment of DN, this paper adopted a focused analysis method and used ClueGO software (Bindea et al., 2009) to analyse the biological processes and KEGG. The common target genes obtained by screening were input into ClueGO software, the GO term fusion was used, and a threshold *p* ≤ 0.05 was set for enrichment.

The GO biological process enrichment parameter "GO Tree Interval" was set to 4–9, the minimum gene of "GO Term Selection" was set to 5, the minimum gene proportion was set to 5%, and the kappa score was set to 0.5. The minimum gene of the KEGG pathway enrichment parameter "GO pathway selection" was 6, with the minimum gene accounting for 4%, and the kappa score was set as 0.5. After the selection parameters were run separately, the GO biological processes and KEGG pathway selection and their related target information were obtained.

### Molecular Docking Verification of Core Compounds and Core Target Genes

First, the top seven core compounds were selected, and the two-dimensional structure diagrams of the compounds were downloaded from the TCMSP database and saved in mol2 format. Then, the files were imported into AutoDockTools-1.5.6 software to add charge and display rotatable keys and then they were saved in pdbqt format. Second, the protein crystal structures corresponding to the core target genes were downloaded from the PDB database (Burley et al., 2017), imported into PyMOL software to remove the water molecules and heteromolecules, imported into AutoDockTools-1.5.6 software to add hydrogen atoms and charge operations, and saved to pdbqt format. The three-dimensional grid box for molecular docking simulation was also obtained using Autodock tools 1.5.6. Finally, AutoDock Vina 1.1.2 (Trott and Olson, 2010) was used to perform molecular docking. The results were analysed and interpreted using PyMOL (DeLano, 2002) and Ligplot (Laskowski and Swindells, 2011) software.

## Results

### Active Components and Corresponding Target Proteins of TW

**FIGURE 1:**
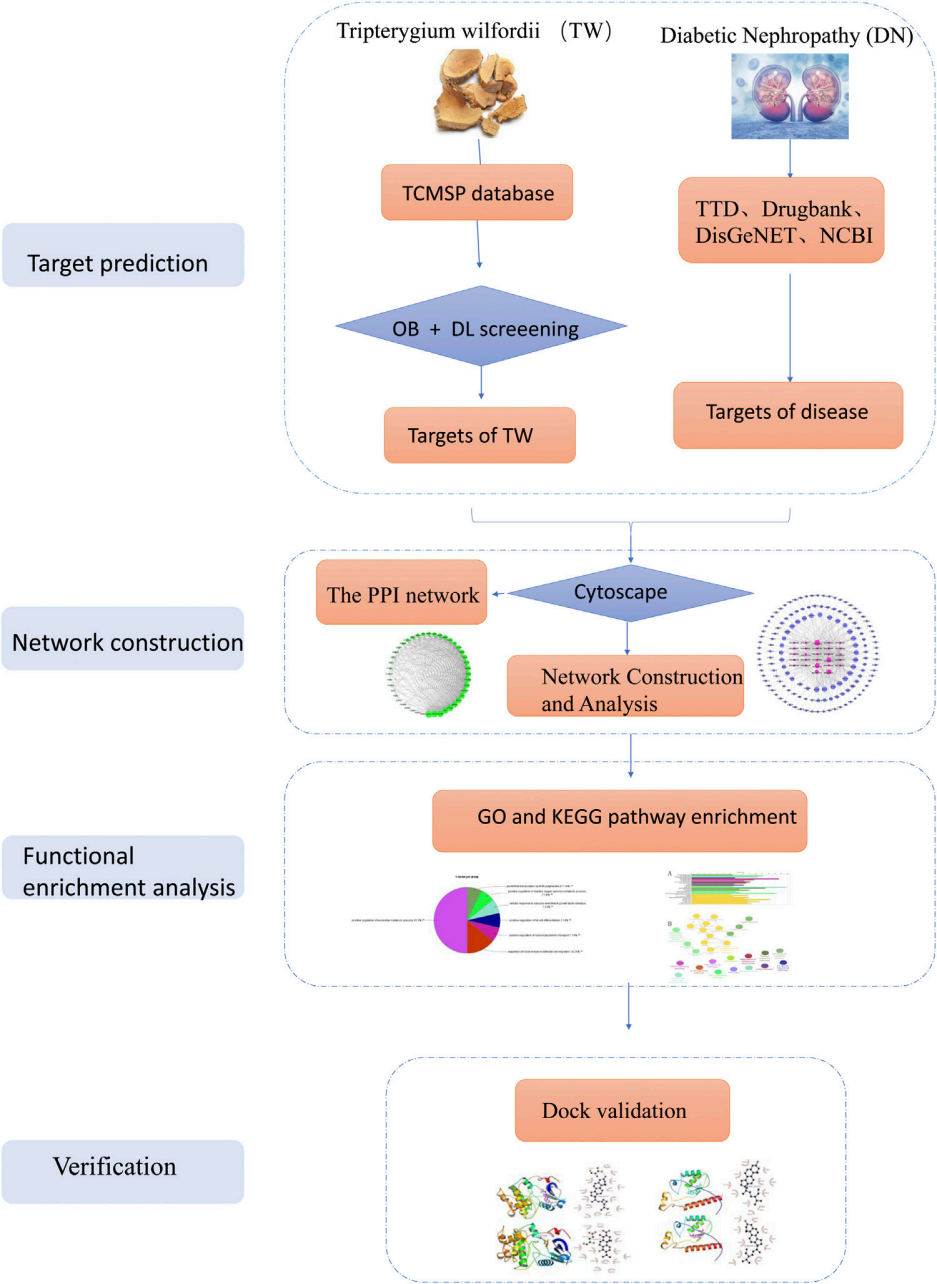
The workflow of the analysis for this study.

**FIGURE 2:**
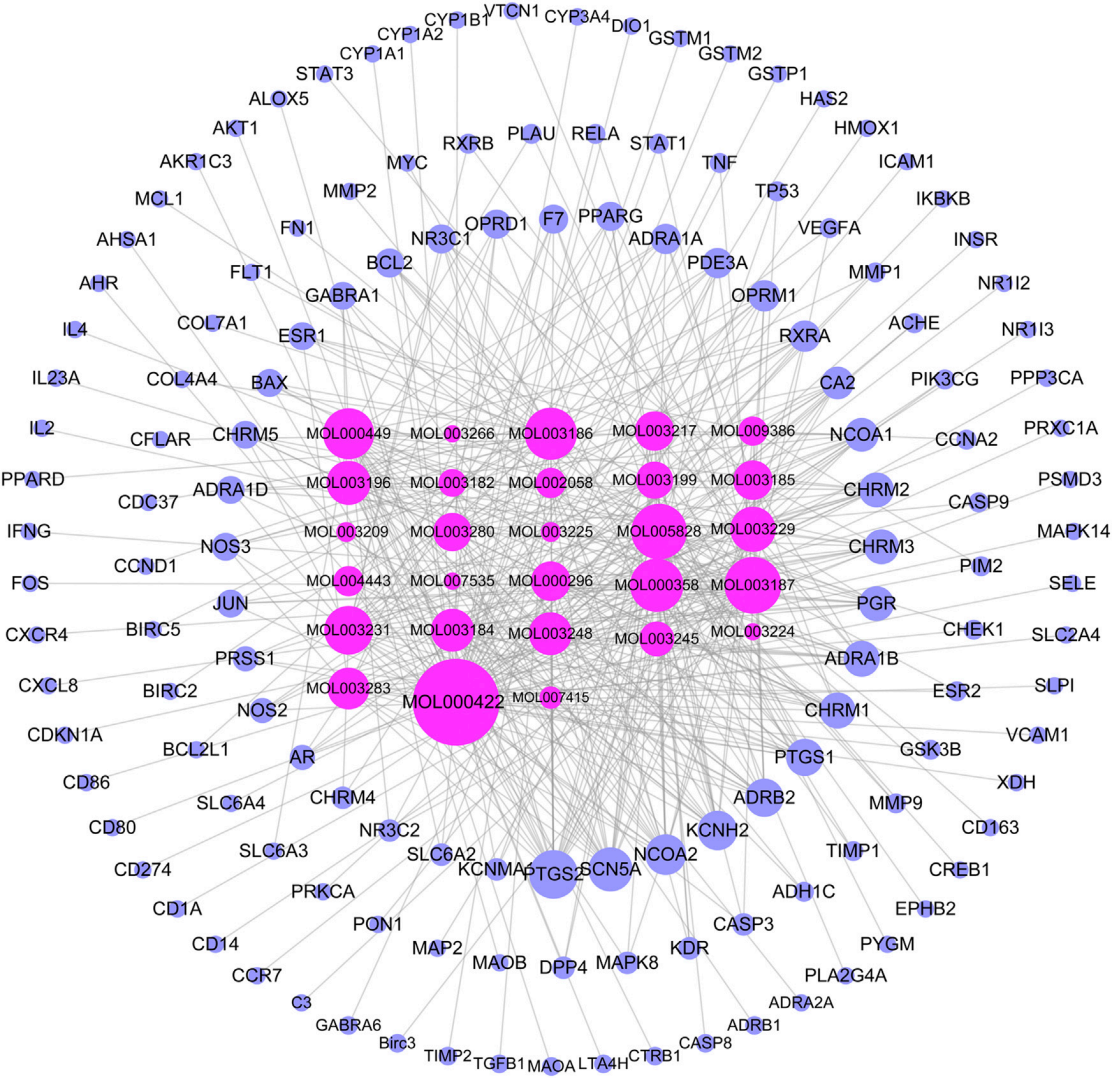
TW- active ingredient-target diagram. The rose red inner circle is the compound (the compound without target is deleted), and the blue outer circle is the target. The larger the node, the greater the degree value, and the closer the prompt relationship.

A total of 144 chemical constituents and 51 active components of TW were screened by TCMSP, mainly alkaloids and terpenoids, as shown in Table 1. To supplement tripterine, although its bioavailability is poor, related experimental studies have shown that it has a significant role in the treatment of DN. The active ingredient-target network diagram is shown in Figure 1 Figure 2. According to the topological parameters, the key compounds of "degree > average value (15.21)" are kaempferol, triptolide, nobiletin, beta-sitosterol, tripterine, stigmasterol, triptoditerpenic acid B, triptinin B, tryptophenolide, 81,827-74-9, triptonoterpene and (2R,3R,4S)-4-(4-hydroxy-3-methoxy-phenyl)-7-methoxy-2,3-dimethylol-tetralin-6-ol, a total of 12 species, which may play a key role in TW treatment of DN.

**TABLE 1:** Active ingredients of TW.

Number	Mol ID	Molecule name	OB (%)	DL
1	MOL004443	Zhebeiresinol	58.72	0.19
2	MOL003267	Wilformine	46.32	0.2
3	MOL003189	WILFORLIDE A	35.66	0.72
4	MOL003196	Tryptophenolide	48.5	0.44
5	MOL003248	Triptonoterpene	48.57	0.28
6	MOL003280	TRIPTONOLIDE	49.51	0.49
7	MOL003245	Triptonoditerpenic acid	42.56	0.39
8	MOL003244	Triptonide	68.45	0.68
9	MOL003192	Triptonide	67.66	0.7
10	MOL003187	Triptolide	51.29	0.68
11	MOL003242	Triptofordinine A2	30.78	0.47
12	MOL003241	Triptofordin F4	31.37	0.67
13	MOL003239	Triptofordin F2	33.62	0.67
14	MOL003238	Triptofordin F1	33.91	0.6
15	MOL003236	Triptofordin D2	30.38	0.69
16	MOL003235	Triptofordin D1	32	0.75
17	MOL003234	Triptofordin C2	30.16	0.76
18	MOL003233	Triptofordin B2	107.71	0.76
19	MOL003232	Triptofordin B1	39.55	0.84
20	MOL003231	Triptoditerpenic acid B	40.02	0.36
21	MOL003229	Triptinin B	34.73	0.32
22	MOL003224	Tripdiotolnide	56.4	0.67
23	MOL003188	Tripchlorolide	78.72	0.72
24	MOL000449	Stigmasterol	43.83	0.76
25	MOL003222	Salazinic acid	36.34	0.76
26	MOL003278	Salaspermic acid	32.19	0.63
27	MOL011169	Peroxyergosterol	44.39	0.82
28	MOL005828	Nobiletin	61.67	0.52
29	MOL000211	Mairin	55.38	0.78
30	MOL000422	Kaempferol	41.88	0.24
31	MOL003217	Isoxanthohumol	56.81	0.39
32	MOL003225	Hypodiolide A	76.13	0.49
33	MOL000296	Hederagenin	36.91	0.75
34	MOL003211	Celaxanthin	47.37	0.58
35	MOL003210	Celapanine	30.18	0.82
36	MOL003209	Celallocinnine	83.47	0.59
37	MOL003208	Celafurine	72.94	0.44
38	MOL003206	Canin	77.41	0.33
39	MOL000358	Beta-sitosterol	36.91	0.75
40	MOL003279	99694-86-7 (15-hydroxytriptolide)	75.23	0.66
41	MOL003184	81827-74-9	45.42	0.53
42	MOL003199	5,8-Dihydroxy-7-(4-hydroxy-5-methyl-coumarin-3) coumarin	61.85	0.54
43	MOL003198	5 Alpha-Benzoyl–4 alpha-hydroxy–1 beta, 8 alpha-dinicotinoyl-dihydro-agarofuran	35.26	0.72
44	MOL002058	40957-99-1	57.2	0.62
45	MOL009386	3,3'-bis-(3,4-dihydro-4-hydroxy-6-methoxy)-2H-1-benzopyran	52.11	0.54
46	MOL003266	21-Hydroxy-30-norhopan-22-one	34.11	0.77
47	MOL007415	[(2S)-2-[[(2S)-2-(benzoylamino)-3-phenylpropanoyl]amino]-3-phenylpropyl] acetate	58.02	0.52
48	MOL007535	(5S,8S,9S,10R,13R,14S,17R)-17-[(1R,4R)-4-ethyl-1,5-dimethylhexyl]-10,13-dimethyl-2,4,5,7,8,9,11,12,14,15,16,17-dodecahydro-1H-cyclopenta[a]phenanthrene-3,6-dione	33.12	0.79
49	MOL003283	(2R,3R,4S)-4-(4-hydroxy-3-methoxy-phenyl)-7-methoxy-2,3-dimethylol-tetralin-6-ol	66.51	0.39
50	MOL003185	(1R,4aR,10aS)-5-hydroxy-1-(hydroxymethyl)-7-isopropyl-8-methoxy-1,4a-dimethyl-4,9,10,10a-tetrahydro-3H-phenanthren-2-one	48.84	0.38
51	MOL003182	(+)-Medioresinol di-O-beta-D-glucopyranoside_qt	60.69	0.62

### Diabetic Nephropathy (DN) related Genes

A total of 755 target genes related to DN were retrieved from the TTD, DrugBank, DisGeNET and NCBI databases (19 were retrieved from TTD, 43 from DrugBank, 560 from DisGeNET, 405 from NCBI, and 272 duplicates were deleted).

### Potential Target of TW in Treating DN

**FIGURE 3:**
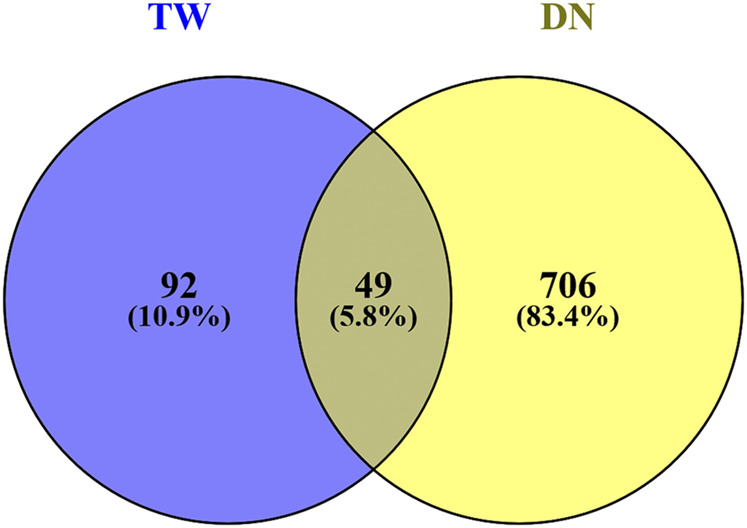
Venn diagram of drugs and disease targets.

The targets of TW and DN were input into a Venn diagram for mapping and intersection, and a total of 49 cross targets were obtained, that is, the potential targets of TW in treating DN, as shown in Figure 3.

### PPI Network Construction and Core Target Screening

**FIGURE 4:**
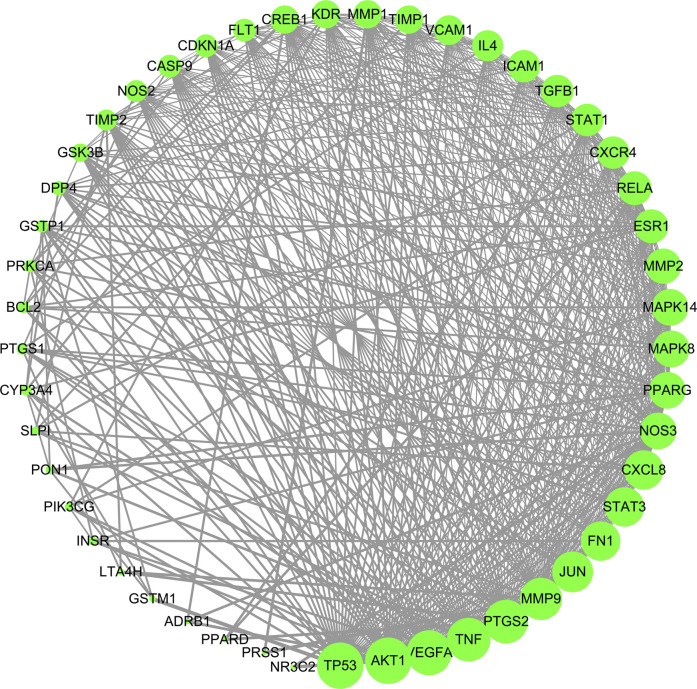
PPI network of TW treating DN. Node degree is positively correlated with node size. The larger the node is, the greater the node degree value is, and the more likely it is to play a role through the target.

The 49 common targets of TW and DN were used to draw protein interaction diagrams through String and Cytoscape software. As shown in Figure 4, the network diagram contains 49 nodes and 524 interaction lines. The node degree is positively correlated with the node size. The larger the node is, the greater the node degree value is, and the more likely it is to play a role through the target. The denser the connection, the more important it is. According to the topological relationship, a total of 16 core targets were selected with "Degree>Mean (21.39) and Betweenness Centrality > Mean (0.0127)", as shown in Table 2.

**TABLE 2:** The core targets of TW in the treatment of DN.

Number	Target gene	Degree	Betweenness Centrality
1	AKT1	38	0.07379271
2	TP53	38	0.06725352
3	VEGFA	37	0.03571233
4	PTGS2	36	0.04609104
5	TNF	36	0.03240009
6	MMP9	35	0.05231183
7	JUN	34	0.01403485
8	FN1	33	0.01425303
9	CXCL8	33	0.01402184
10	NOS3	31	0.04440636
11	PPARG	31	0.02808594
12	RELA	29	0.01695853
13	ESR1	29	0.0150523
14	STAT1	28	0.01927069
15	MMP1	25	0.01576515
16	CREB1	25	0.01447089

### Results of Biological Process Enrichment Analysis

**FIGURE 5:**
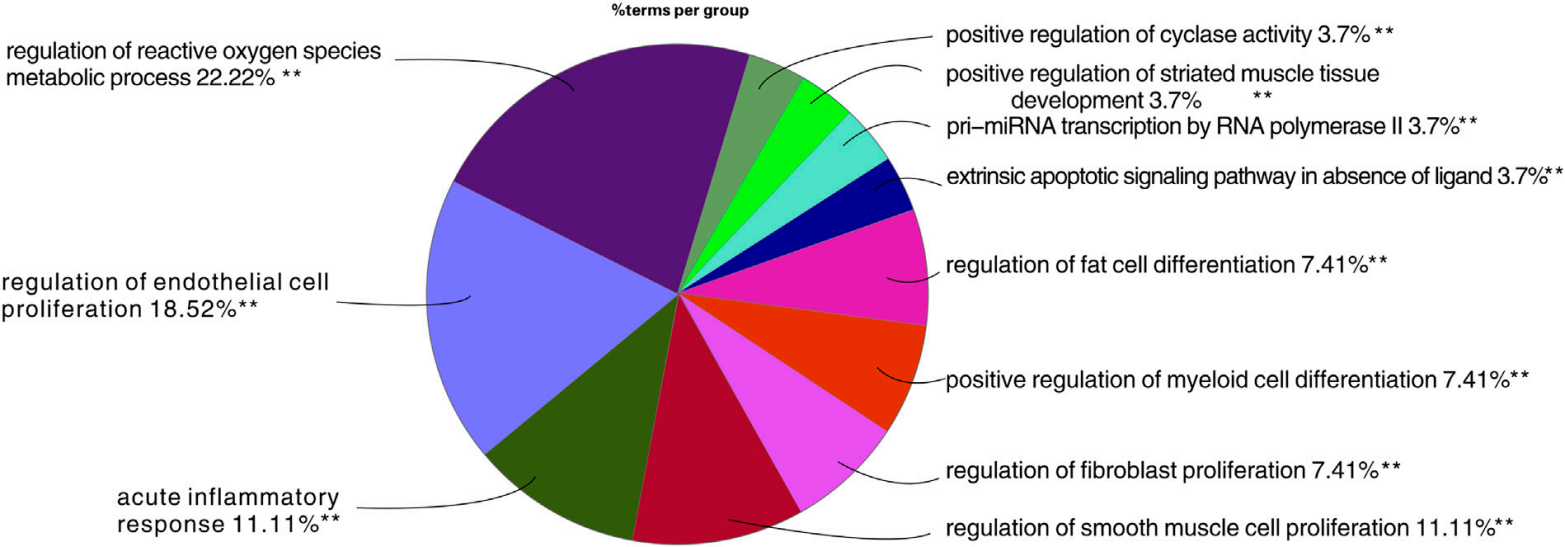
Results of GO analysis TW treating DN. The larger the area in the figure, the more mapping targets clustered to this biological function.

Through GO biological process analysis, 27 major biological processes of TW treating DN were obtained, mainly enriched in 11 categories, as shown in Figure 5. The larger the area in the figure, the more mapping targets clustered to this biological function. From the figure, it can be inferred that the treatment of DN by TW may be mainly related to the following biological processes: ① regulation of oxygen species metabolic process 22.22%; ② regulation of endothelial cell proliferation 18.52%; ③ acute inflammatory response 11.11%; ④ extrinsic apoptotic signaling pathway in the absence of ligand 3.7%; ⑤ regulation of fibroblast proliferation 7.41%; ⑥ positive regulation of cyclase activity 3.7%; ⑦ regulation of fat cell differentiation 7.41%, etc.

### Results of the KEGG Cluster Analysis

**FIGURE 6:**
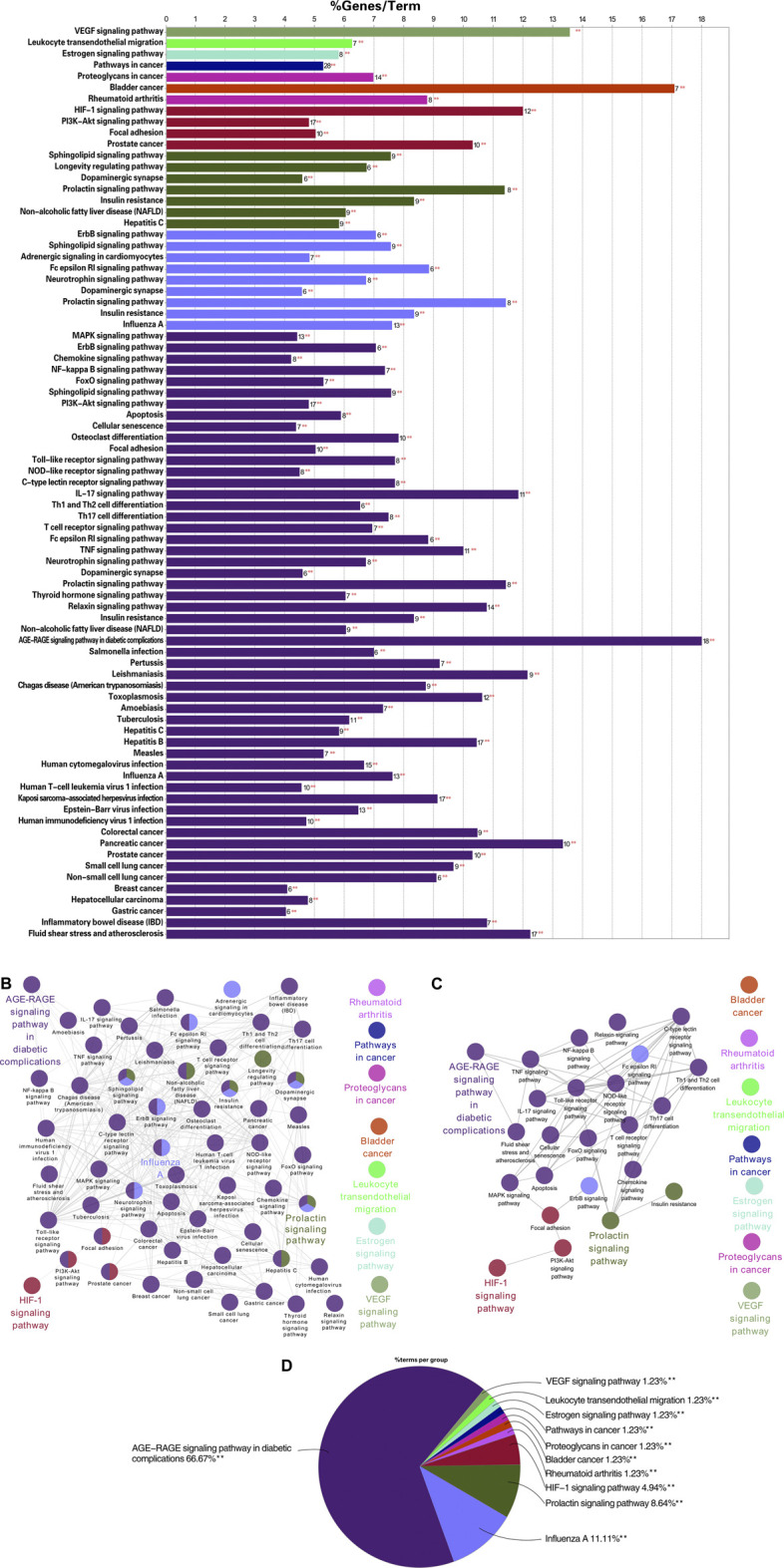
KEGG enrichment analysis results of TW-treated DN (**6A**) the KEGG pathway of TW in the treatment of DN. The horizontal axis represents the proportion of gene enrichment, the vertical axis represents different pathways, and the number on the bar chart is the number of target genes in this pathway. (**6B**) The relationship between the pathways with a significant degree of enrichment. The same color indicates biological processes with similar functions, and the bolded labels indicate pathways with a significant degree of enrichment (**6C**) The enrichment of pathways closely related to the treatment of DN by TW was further screened on the basis of B6. (**6D**) The proportion of each enrichment result.

To further explore the specific molecular mechanism of TW in the treatment of DN, the KEGG pathway was explored. As shown in Figures 6A A total of 64 signalling pathways were obtained (among them, 25 were closely related to DN,6 were related, and a total of 31 were involved). The percentage of hit genes and the gene composition of these 31 related pathways are shown in Supplementary Table S1, where the horizontal axis represents the proportion of gene enrichment, the vertical axis represents different pathways, and the number on the bar chart is the number of target genes in this pathway. The relationship between the pathways with a significant degree of enrichment is shown in Figure 6B. The same colour indicates biological processes with similar functions, and the bolded labels indicate pathways with a significant degree of enrichment. According to relevant research progress, 25 pathways closely related to DN were extracted on the basis of the enrichment in Figure 6B, as shown in Figure 6C. The proportion of each enrichment result is shown in Figure 6D. The pathways enriched in AGE-RAGE were the most enriched, followed by HIF-1, VEGF, leukocyte transendothelial migration, rheumatoid arthritis, and the estrogen signaling pathway.

### Molecular Docking Verification of Core Compounds and Core Target Genes

**FIGURE 7:**
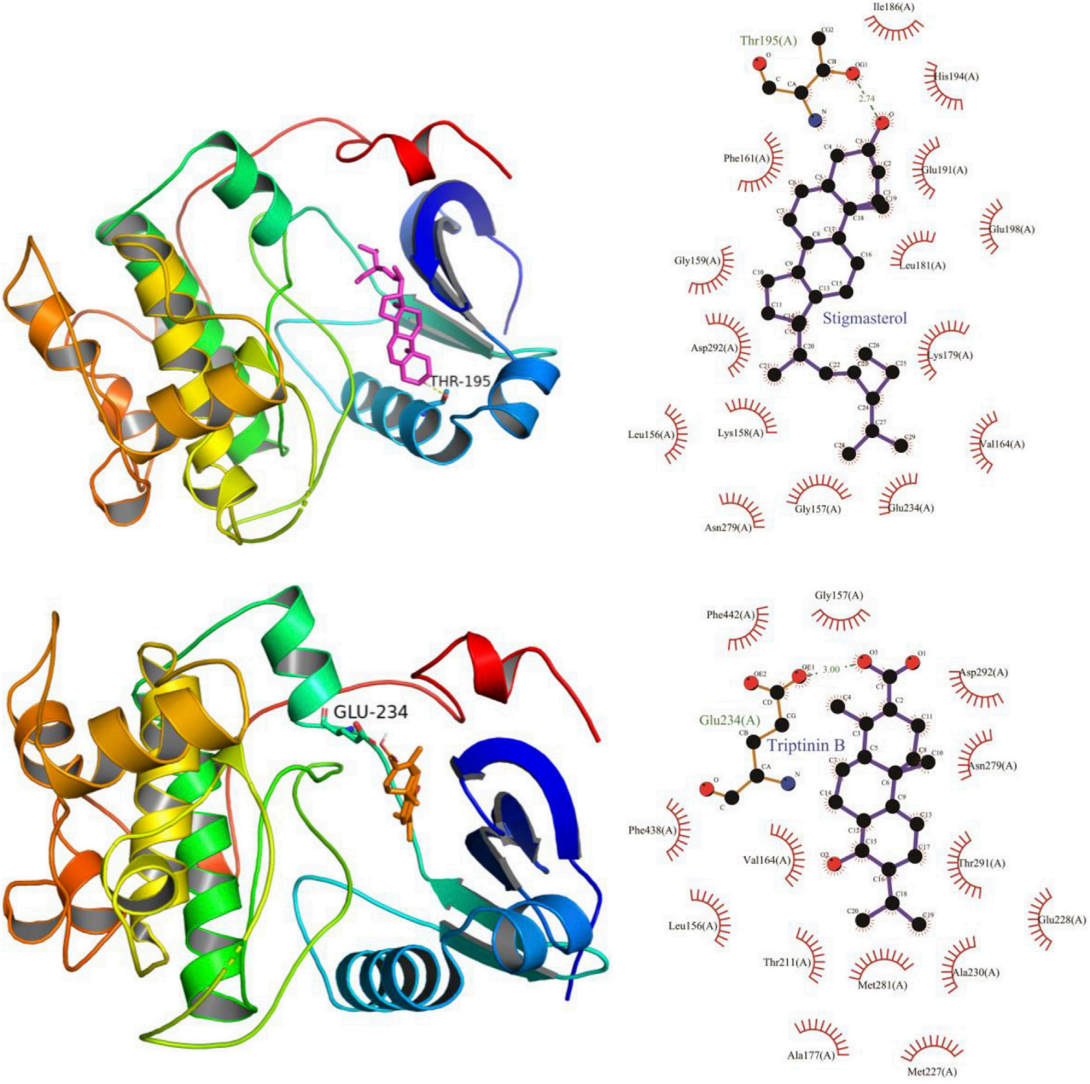
Molecular docking diagram of TW core compounds and AKT1.

**FIGURE 8:**
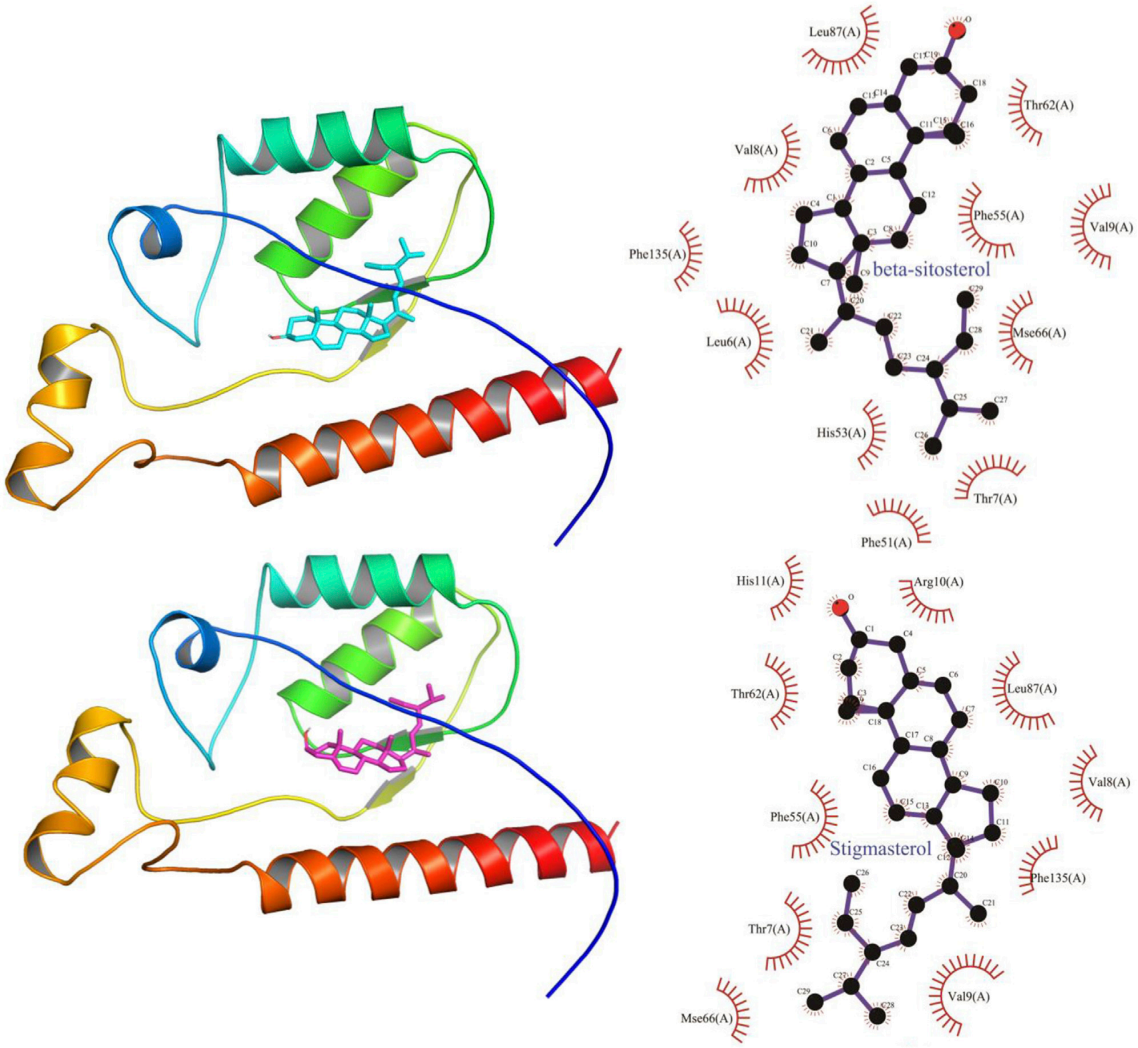
Molecular docking diagram of TW core compounds and TP53.

**FIGURE 9:**
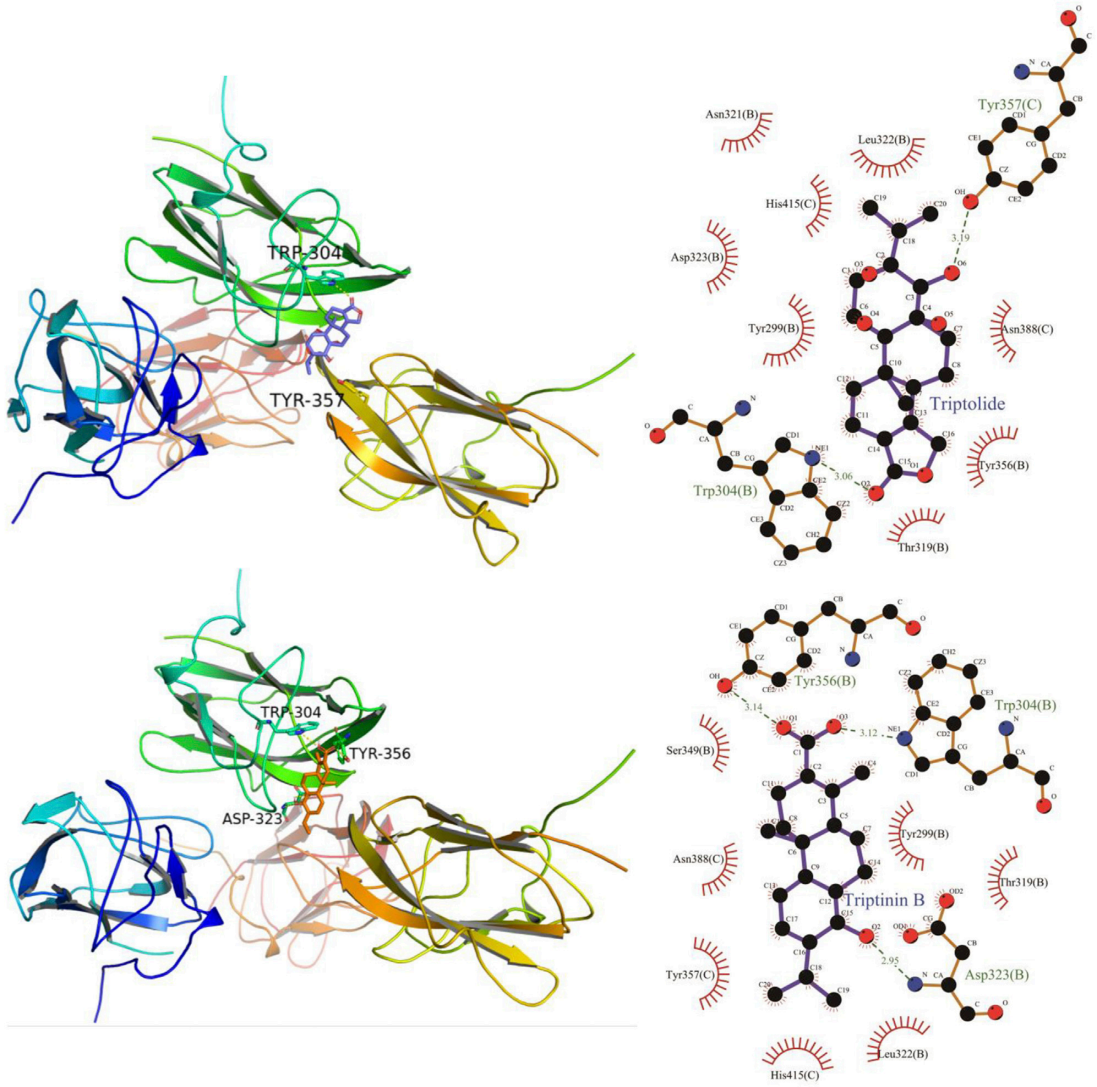
Molecular docking diagram of TW core compounds and VEGF.

The results obtained by the molecular docking software are shown in Table 3. The grid box was centered to cover the active binding site and all essential residues. For AKT1, the grid box (44 Å × 40 Å × 54 Å) was centred at (2.878, –0.196, 25.87) Å, for VEGF, the grid box (72 Å × 78 Å × 88 Å) was centred at (13.697, 45.246, –1.911) Å, and for TP53, the grid box (40 Å × 46 Å × 50 Å) was centered at (27.292, 35.046, 3.963) Å. As seen from Table 3, the scores for the five core compounds (kaempferol, triptolide, nobiletin, beta-sitosterol, stigmasterol, triptoditerpenic acid B, and triptinin B) and protein crystal structures corresponding to the core target genes (AKT1, TP53, and VEGFA) were all greater than −5 kcal/mol, indicating that the compound had a certain affinity for the protein crystal structure. Molecular docking was performed to determine the best candidates among the 9 phytochemicals based on their binding scores. Stigmasterol and triptinin B show the highest binding affinities of –9.3 and –8.7 kcal/mol for AKT1, beta-sitosterol and stigmasterol showed the highest binding affinities of –9.5 and –9.3 kcal/mol for TP53, and triptolide and triptinin B showed the highest binding affinities of –9.0 and –8.5 kcal/mol for VEGFA. Figures 7, 8 and 9 show that the small-molecule compounds were tightly bound to the protein residues via various interactions.

**TABLE 3:** The binding energy values of core compounds of TW and core targets.

Target	Compounds	Binding energy/(kcal/mol)
AKT1(3cqw)	Kaempferol	–8.1
AKT1(3cqw)	Triptolide	–8.1
AKT1(3cqw)	Nobiletin	–7.7
AKT1(3cqw)	Beta-sitosterol	–8.2
AKT1(3cqw)	Tripterine	–8.7
AKT1(3cqw)	Stigmasterol	–9.3
AKT1(3cqw)	Triptoditerpenic acid B	–8.1
AKT1(3cqw)	Triptinin B	–8.7
TP53(3dcy)	Kaempferol	–8
TP53(3dcy)	Triptolide	–8.3
TP53(3dcy)	Nobiletin	–8.9
TP53(3dcy)	Beta-sitosterol	–9.5
TP53(3dcy)	Tripterine	–8.6
TP53(3dcy)	Stigmasterol	–9.3
TP53(3dcy)	Triptoditerpenic acid B	–7.6
TP53(3dcy)	Triptinin B	–8.3
VEGFA(5dn2)	Kaempferol	–7.8
VEGFA(5dn2)	Triptolide	–9
VEGFA(5dn2)	Nobiletin	–7.3
VEGFA(5dn2)	Beta-sitosterol	–7.5
VEGFA(5dn2)	Tripterine	–7.7
VEGFA(5dn2)	Stigmasterol	–7.7
VEGFA(5dn2)	Triptoditerpenic acid B	–7.6
VEGFA(5dn2)	Triptinin B	–8.5

## Discussion

TW is a promising traditional Chinese medicine, which can significantly reduce proteinuria and improve renal function. However, its toxicity limits its clinical application. With the development of various biotechnologies, TW and its extracts have been recognized as a key alternative intervention measures for the treatment of DN in the 2014 Consensus on Prevention of Diabetic Nephropathy in China, and its efficacy has been confirmed in many experiments (Huang et al., 2020). Evidence-based medicine research shows that TW can effectively reduce proteinuria, serum creatinine, and blood urea nitrogen levels, and increase the effective rate (Hong et al., 2016; Wang D. et al., 2018). *In vivo* experimental studies which shows that TW extract can improve the micro-inflammatory state of DN (Wu et al., 2017), anti-oxidative stress, reduce proteinuria, improve glomerular hypertrophy and podocyte injury, and alleviate renal fibrosis (Song et al., 2019), and it is even superior to other drugs in anti-inflammatory and oxidative stress (Gao et al., 2010; Ma et al., 2013). *In vitro* experimental studies, it is shown that triptolide, an extract of TW, has effects of anti-inflammatory, anti-oxidative stress, protection and reversal podocyte injury, and maintenance of podocyte filter barrier function (Zheng et al., 2008; Chen et al., 2010; Song et al., 2019; Wang et al., 2019; Liang et al., 2020). Although TW and its extracts have positive effects in the treatment of DN, we should also pay attention to its side effects. According to reports, TW has gastrointestinal reactions, liver damage, menstrual disorders, reproductive problems, adverse skin reactions, hematological events, cardiovascular events and nephrotoxicity (Zhang C. et al., 2016; Brown, 2017). Therefore, researchers should devote themselves to finding bioactive substances with safe doses and modifying their structures; reasonable combination of drugs; purifying their extracts; optimizing extraction methods; changing their dosage forms and administration methods, reduce the toxicity, so as to make TW better serve the clinic (Shao et al., 2006; Hewitson et al., 2009; Tang and Zuo, 2012; Zhang et al., 2014; Yuan et al., 2015; Zhang B. et al., 2016; Li Z. et al., 2017).

In this study, 151 compounds of TW were obtained through preliminary screening by TCMSP, 51 active ingredients were obtained by further screening according to the ADME parameters, and 1 was supplemented by the literature. A total of 141 potential targets were identified, and 755 DN-related targets intersected. Finally, 49 common targets were obtained. These targets were mainly focused on AGE-RAGE, vascular endothelial growth factor (VEGF), HIF-1, IL-17, the relaxin signaling pathway, TNF, Fc epsilon RI, insulin resistance and other signaling pathways.

### Discussion of Key Active Ingredients and Core Targets

The active component-target network diagram and related topological parameters show that the key active ingredients of TW are kaempferol, triptolide, nobiletin, and tripterine. TW mainly improves kidney damage and alleviates related symptoms by regulating oxidative stress, cell apoptosis, insulin resistance, etc. The 12 key compounds were mainly terpenoids, among which kaempferol and caseolin were flavonoids. Kaempferol has anti-inflammatory, antioxidant and lipolysis effects (Zhang et al., 2013). Related studies have shown that kaempferol can inhibit the oxidative stress and apoptosis of human glomerular endothelial cells induced by high glucose (Sharma et al., 2019). Nobiletin has biological effects such as anti-inflammatory and antioxidative effects, lowering blood pressure, lowering cholesterol, and changing the local microcirculation. Related research reports (Lee et al., 2010) have shown that nobiletin can reduce insulin resistance and blood lipids in obese type 2 diabetic rats. In addition, Malik et al. (2015) found that nobiletin can improve acute kidney injury. Triptolide and tripterine are terpenoids. Triptolide has anti-inflammatory effects (Ma et al., 2013), regulating oxidative stress (Gao et al., 2010; Dong et al., 2017), anti-fibrosis (Li X.-Y. et al., 2017; Li et al., 2019) and anti-glomerular sclerosis (Han et al., 2017; Han et al., 2018). It has significant effects on the treatment of proteinuria, reducing podocyte damage (Ma et al., 2013), reducing the accumulation of DN mesangial matrix and alleviating mesangial dilation (Han et al., 2017). In addition, the results of molecular docking and kinetic simulation showed that triptolide has a similar structure to hormones and can bind to nuclear receptors (Liu et al., 2015). Tripterine, also known as celastrol, is a quinone methide triterpene (Yang et al., 2006). Although its oral bioavailability is low, it can reduce oxidative stress damage and podocyte depletion caused by high sugar. It can also reduce insulin resistance (Kim et al., 2013), reduce inflammation (Zhou and Huang, 2009; Lee et al., 2015; Zhang M. et al., 2019), and restore autophagy pathways impaired by high glucose (Zhan et al., 2018). In addition, tripterine can also prevent renal injury caused by ischaemia reperfusion (Kim et al., 2013; Chu et al., 2014). Previous studies on the pharmacodynamics of TW have mainly focused on alkaloids and terpenoids, and there have been few studies on flavonoids. The results of this study showed that the key compounds of TW, kaempferol and rhizopetin, were flavonoids, which provided a new direction for clinical research.

There were 16 core targets screened through PPI, such as AKT1, TP53, VEGFA, PTGS2, TNF, MMP9, JUN, FN1, CXCL8, NOS3, PPARG, RELA, ESR1, STAT1, MMP1, and CREB1, which may play a key role in the treatment of DN.

In this study, it was found that AKT1 was directly linked to 51 of the 64 screened pathways, which may be one of the important targets for TW to regulate multiple pathways. AKT1 is a serine/threonine kinase that can regulate insulin metabolism. In addition, AKT1 is an important factor in the PI3K/AKT pathway and it plays an important role in regulating glucose homeostasis, lipid metabolism, protein synthesis, and cell survival (Fischer-Posovszky et al., 2012; Huang et al., 2018). Further studies (Heljić and Brazil, 2011) showed that AKT1 is an important regulator of TGF-β1-mediated biological processes, and it can stimulate the regulatory transduction systems in renal cells, such as Smad and mTOR, and regulate various cells, such as popopocytes, mesangial cells and renal tubular epithelial cells.

Targets such as VEGFA, CXCL8, and TNF are all proinflammatory factors, and they jointly participate in the chronic inflammatory response process of DN. Among them, VEGFA is the main contributor to the development of new blood vessels, and it is also a proinflammatory cytokine (Fatima et al., 2017), which is closely related to kidney inflammation (Lavoz et al., 2020). It is a protein secreted by podocytes and is necessary for the survival of endothelial cells, podocytes and mesangial cells (Tufro and Veron, 2012). VEGF-A regulates the signal transduction of the slit membrane and the shape of the podocyte through its interaction with the VEGF receptor 2-nephrin-nck-actin, which is essential for maintaining the glomerular filtration barrier (Tufro and Veron, 2012). In the diabetic state, VEGF is significantly upregulated and it participates in podocyte pathology, especially proteinuria (Hanefeld et al., 2016; Bus et al., 2017; Lavoz et al., 2020). Animal studies have shown that when inhibiting the activity of VEGF, proteinuria is significantly improved (Vriese et al., 2001; Flyvbjerg et al., 2002; Sung et al., 2006). The combination of CXCL8 and its CXC chemokine receptors (CXCR1 and CXCR2) can recruit neutrophils to infiltrate and induce tissue inflammation. Studies (Cui et al., 2017) have proven that blocking CXCR 1/2 can alleviate diabetic mouse kidney inflammation and renal fibrosis. As a proinflammatory factor, TNF is involved in the process of the chronic inflammatory response in DN (Navarro and Mora-Fernández, 2006; Sun and Kanwar, 2015).

Both NOS3 and PTGS-2 are enzymes that participate in the inflammatory response by catalysing inflammatory factors. NOS3 catalyses the production of nitric oxide (NO), which is closely related to endothelial cell function, and vascular endothelial dysfunction has been considered an important factor in the pathogenesis of DN (Zeng et al., 2010). Other studies have shown that the polymorphism of the NOS3 gene is related to the rapid deterioration of renal function in CKD patients (Medina et al., 2018); PTGS-2 is a key enzyme in the initiation of prostaglandin synthesis *in vivo* and is a major target of NSAIDs for treatment (Vane, 1971; Flower, 1974). It mainly causes diabetic renal damage by mediating inflammation and affecting renal haemodynamics (Morham et al., 1995; Martín-Sanz et al., 2006). In addition, PTGS-2 is responsible for maintaining kidney homeostasis function and it is involved in salt absorption, fluid regulation volume and blood pressure (Martín-Sanz et al., 2006).

Both fibronectin (FN) and matrix metalloproteinases (MMPs) play a role in the treatment of DN by affecting extracellular matrix (ECM) proteins. The early pathological features of DN are glomerular hypertrophy, basement membrane thickening and mesangial dilatation, and late stages involve glomerular sclerosis and interstitial fibrosis. Excessive accumulation of ECM is the common pathological basis and it eventually causes glomerular sclerosis. FN, an important component of the ECM, is distributed in the glomerular basement membrane, mesangial membrane and plasma. The abnormal increase in fibronectin in the ECM plays an important role in the pathogenesis of DN (Eddy, 1996; Sharma et al., 1996). MMPs affect the decomposition and conversion of ECM proteins (Li et al., 2014). In addition, "*in vitro*" and animal studies have shown that renal fibrosis is positively correlated with the expression and activation of MMPs, and urine MMP-1, -2, -9 excretion and the urine MMP-1, -2, -9/TIMP–1 ratio can be used as early biomarkers of renal fibrosis (Ahmed et al., 2007; Hirt-Minkowski et al., 2014; Bieniaś and Sikora, 2018). CREB1 is closely related to FN. In the early stage of DN, CREB1 increases the production of FN by binding to the promoter of the fibronectin gene, leading to the accumulation of FN, decreased glomerular sclerosis filtration (Singh et al., 2001), and renal tubulointerstitial fibrosis (Visavadiya et al., 2011). CREB1 is overactivated during diabetes, leading to fasting hyperglycaemia. Blocking the expression of enzymes by CREB is another strategy for the treatment of diabetes and its complications (Benchoula et al., 2021).

STAT1, RELA, JUN, are all transcription factors. STAT1 is a member of the STAT family and it acts as a signaling messenger and transcription factor (Begitt et al., 2014). It regulates the expression of genes related to cell proliferation, oxidative stress and apoptosis (Bowman et al., 2000; Ramana et al., 2000; Stark and Darnell, 2012). In the inactivated state, STAT1 can reverse podocyte injury triggered by high glucose and it plays a key role in renal fibrosis and apoptosis (Huang et al., 2019). There are also related reports that STAT1 knockdown can inhibit cell death, which highlights the importance of STAT1 as a new treatment for renal fibrosis (Wang S. et al., 2018); RELA and JUN are involved in the inflammatory response and renal fibrosis in the process of DN, and their activity can be mediated by the MAPK pathway (Turpaev, 2006; Sanchez and Sharma, 2009).

PPARG involves the expression of various genes, such as insulin sensitivity, and it plays an important role in glucose and lipid metabolism (Kroker and Bruning, 2015; Ya-Fei, 2019). Recent studies (Kroker and Bruning, 2015) have shown that some PPARG agonists retain insulin sensitization with few side effects and are widely used in the treatment of type 2 diabetes mellitus; ESR includes ESR1 and ESR2, and most of the effects of oestrogen are mediated by ESR1. The combination of oestrogen and ESR1 can reduce the synthesis of angiotensin 2 and endothelin, thereby inhibiting renal vasoconstriction and reducing renal inflammation (Bupp, 2015). In addition, oestrogen can also promote the expression of MMP-2 and stimulate the synthesis of MMP-9, thereby reducing the level of endothelial cell fibrosis and improving DN (Guccione et al., 2002). TP53 is an important tumour regulatory gene. There are few studies related to DN at present, but it may be a new research direction. The acquisition of these target proteins provides a reference for the clinical diagnosis and treatment of DN.

### Discussion of Signalling Pathways

The results of KEGG pathway enrichment analysis showed that the pathways of TW in treating DN were mainly enriched in five categories: inflammatory response, oxidative stress and immune regulation, anti-vascular disease, insulin resistance, renal fibrosis and apoptosis.

First, the inflammatory response, oxidative stress and immune regulation: Although the pathogenesis of DN is not yet fully understood, it is certain that oxidative stress, inflammation, and immunity play an important role in the occurrence and development of DN (Zhang B. et al., 2019). Inflammation is considered to be an important mechanism in the pathogenesis of DN, mediated by oxidative stress, transcription factors (including nuclear factor κB (NF-κB) and inflammatory cytokines (including Toll-like receptors, chemokines, plasma molecules and proinflammatory cytokines). In addition, activated innate immunity and inflammation are closely related to the pathogenesis of DN (Baelde et al., 2004). ① NF-κB signalling pathway: When excessive reactive oxygen species (ROS) are produced in the kidney tissue of diabetic patients, oxidative stress occurs (Sharma et al., 2019), and the excessive accumulation of oxidized products and unbalanced scavenging ability cause changes in the level of oxidative stress, and then activate the nuclear factor NF-κB. NF-κB is the core factor of inflammation. Activated NF-κB is phosphorylated and transferred from the cytoplasm to the nucleus, causing an inflammatory response, which in turn leads to DN (Yao et al., 2019). The NF-κB pathway not only plays an important role in renal injury but is also one of the most important pathways in improving the podocyte migration induced by high glucose, decreasing the podocyte protein flow rate, and protecting podocyte filtration barrier function (Xiao-wen, 2016). ② AGE-RAGE signaling pathway: The advanced glycosylation end products (AGEs)-receptor of AGEs (RAGE) signalling pathway is an important part of the development of DN and can cause chronic inflammation and oxidative stress in renal tissues. In addition, RAGE activation leads to the activation of different intracellular signalling pathways, such as PI3K/Akt, MAPK/ERK and NF-κB. At present, blocking the formation of the AGE–RAGE axis has become a new treatment strategy (Sanajou et al., 2018). ③ HIF-1 signalling pathway: Hypoxia inducible factor 1 (HIF-1), a transcription factor, is also the only specific transcription factor that can exert biological activity under hypoxic conditions (Zhang et al., 2017). It can be used as the main regulator of oxygen homeostasis to help cells adapt to hypoxic environments and prevent cell damage caused by hypoxia (Richard et al., 2000; Haddad and Land, 2001; Fukuda et al., 2002). ④ The TNF pathway can promote the adhesion and aggregation of inflammatory factors, induce an inflammatory response, participate in microvascular lesions, and eventually damage glomerular tissue (Rakitianskaia et al., 2013). Tumour necrosis factor (TNF-a) in this pathway is a cytokine with a significant proinflammatory effect. It can cause toxic damage, apoptosis and cell necrosis of kidney cells (Laster et al., 1988; Bertani et al., 1989; Boyle et al., 2003). In addition, TNF-a does not rely on haemodynamic mechanisms to promote the production of reactive oxygen species, which ultimately leads to changes in the glomerular capillary wall and increased permeability of albumin (McCarthy et al., 1998). ⑤Leukocyte transendothelial migration: Different types of activated white blood cells play a crucial role in the pathogenesis of most kidney diseases, from acute to chronic. It has been reported that intercellular adhesion molecule 1 and chemokines CCL2 and CX3CL1 may be involved in leukocyte migration in DN (Galkina and Ley, 2006). ⑥ PI3K-Akt, FoX0 and ErbB signalling pathways: The PI3K-Akt signalling pathway is a key pathway for inhibiting cell apoptosis (Rogacka et al., 2014). It participates in the TGF-3 mediated oxidative stress response of glomerular mesangial cells with the FoX0 pathway (Kato et al., 2006), and the ErbB pathway is an important upstream component of the PI3K-Akt pathway (Pan and Dobrowsky, 2013). ⑦ IL-17, NF-κB, VEGF, and chemokines are involved in the chronic inflammatory response, and their mediated pathways may cause damage to glomerular endothelial cells and vascular endothelial function (García-García et al., 2014). ⑧ The T cell receptor, Toll-like receptor (TLR) (Lin et al., 2012) , NOD-like receptor (NLR) (Nielsen et al., 2017), C-type lectin receptor (CLR), FcεRI (Horn et al., 2001; Hamdy et al., 2018) and other immune-related pathways are also closely related to inflammation (Wada and Makino, 2016; Sharma et al., 2018). Damaged kidney cells can trigger an immune system response, activate a variety of immune pathways, promote the synthesis of inflammatory cytokines, trigger chronic inflammation in the kidney, and lead to the occurrence and progression of DN. ⑨ MAPK pathway: The long-term high glucose state can also activate the MAPK pathway, which can cause or accelerate the progression of DN by participating in the processes of renal cell apoptosis, transdifferentiation and immune inflammatory response under stress (Elsherbiny and Al-Gayyar, 2013).

Second, improving vascular disease: DN is a progressive microvascular complication caused by diabetes (Fox et al., 2005) and is generally considered to be the result of the interaction between haemodynamics and metabolic factors (García-García et al., 2014). ① VEGF signalling pathway: VEGF can promote the proliferation of vascular endothelial cells, angiogenesis, and vasodilation and increase vascular permeability (Hyder et al., 1998; Leung et al., 1989); VEGF is also a potential mediator of glomerular filtration and proteinuria. On the one hand, excessive production of VEGF-A by diabetic podocytes in an environment of low endothelial NO is considered to be the main driving force of DN (Takahashi et al., 1998; Tufro and Veron, 2012). On the other hand, in the residual kidney model, VEGF treatment reduced the development of primary glomerulosclerosis and interstitial fibrosis (Kang et al., 2001). In the type 2 experimental model, VEGF antibody treatment improved both the typical early characteristics of DN and late renal changes (Flyvbjerg et al., 2002). ② Relaxin signalling pathway: the relaxin pathway has the effects of relaxing blood vessels, regulating extracellular matrix, and anti-fibrosis and angiogenesis activities (Xie et al., 2015); and ③ the fluid shear stress and atherosclerosis signalling pathway.

Third, insulin resistance: Insulin resistance signalling pathway: Insulin resistance (IR) is an independent risk factor for the occurrence and development of DN (Tahara and Takasu, 2018). In the early stage of DN—microalbuminuria (MA), studies (Tucker et al., 1992) have shown that in patients with type 2 diabetes, the diagnosis of hyperinsulinemia may lead to hyperfiltration and trigger MA. With the further development of DN, IR worsens, and among other factors, it may accelerate the decline of renal function to end-stage renal disease (ESRD) (Svensson and Eriksson, 2006). Jung Eun Kim et al. showed through animal experiments that improving insulin resistance in db/db mice can protect kidney function (Kim et al., 2013).

Fourth, renal fibrosis: The formation mechanism of renal fibrosis is mainly divided into transforming growth factor expression, extracellular matrix deposition, epithelial mesenchymal transformation, inflammatory response, and oxidative stress response (Eddy, 2000; Meng et al., 2014; Sutariya et al., 2016). The NF-κB pathway is a widely studied inflammatory pathway associated with renal fibrosis. PI3K-Akt and HIF-1 play a key role in epithelial-mesenchymal transformation (Higgins et al., 2007; Liu et al., 2016). ① HIF-1 signalling pathway: Hypoxia is considered to be an important microenvironmental factor in the development of tissue fibrosis. Under the long-term high glucose load of DN, the oxygen consumption of renal tissue increases (Takiyama and Haneda, 2014), while the formation of renal interstitial fibres induced by chronic hypoxia are mainly mediated by HIF-1 (Pan et al., 2013; Liu et al., 2017). It consists of two subunits: HIF-1alpha (HIF-1a) and HIF-1beta (HIF-1b). Under hypoxia, HIF-1α induces the upregulation of p53, inhibits the progression of the cell cycle, leads to the accumulation of G2/M cells, activates the fibrotic TGF-β and CTGF-mediated signalling pathways, leads to the production of extracellular matrix, and promotes renal tubulointerstitial fibrosis (Kietzmann et al., 1999; Higgins et al., 2003; Liu et al., 2017; Liu et al., 2019). ② PI3K-Akt is an important factor involved in causing kidney damage in DN (Liu et al., 2016). Blocking the PI3K/AKT pathway in db/db mice can reduce tubular interstitial fibrosis (Yiu et al., 2018). ③ Focal adhesion (FA): FA is an important mediator of the interaction between the endothelial cytoskeleton and ECM transmembrane receptors, integrins and integrin-related intracellular proteins. The role of endothelial FA in diabetic nephropathy has only recently been studied (Infusino and Jacobson, 2012; Elad et al., 2013). Decreasing the expression of FA can reduce the risk of renal fibrosis (Yan et al., 2019). In addition, preventing the reduction of focal adhesions can reduce the loss of podocytes (Yan et al., 2019). FA is also a recognized therapeutic target for proteinuria nephropathy (Yan et al., 2019).

Fifth, apoptosis: TW regulates the cell cycle by regulating apoptosis and cellular senescence pathways, thereby playing a role in the treatment of DN.

**FIGURE 10:**
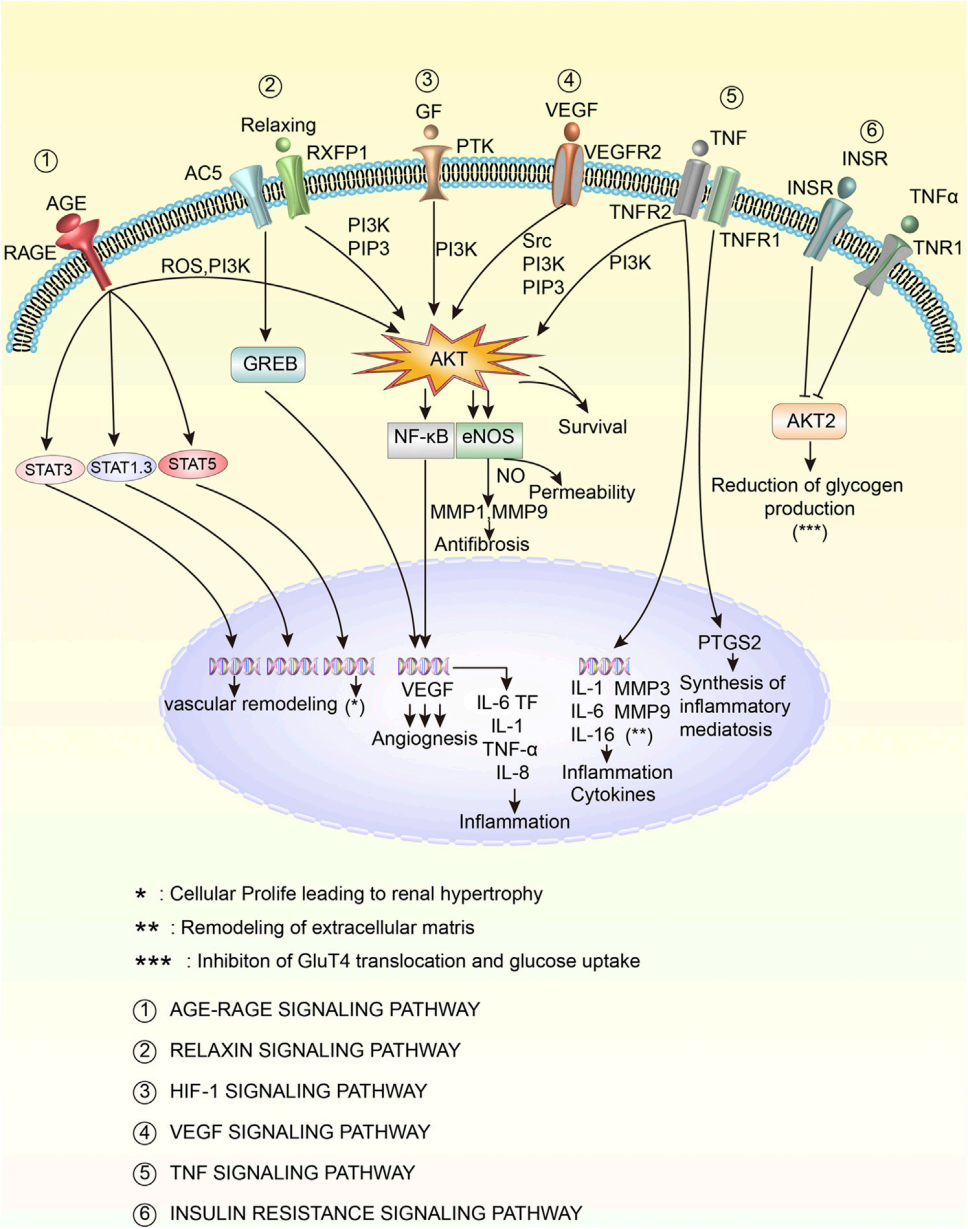
Key mechanisms of TW in the treatment of DN patients. The activation is marked with an arrow, and the inhibition is marked with a vertical line at the top of the arrow.

Further analysis of the enrichment pathways revealed that the top 9 pathways with the highest proportion of hit genes were AGE-RAGE (18.00%), VEGF (13.56%), HIF-1 (12.00%), fluid shear stress and atherosclerosis (11.43%), IL-17 (11.83%), relaxin (10.77%), TNF (10.00%), Fc epsilon RI signaling pathway (8.82%), and insulin resistance (8.33%). The age-rage, VEGF, HIF -1, relaxin, TNF, and insulin resistance pathways were selected to draw the cell pathway diagram, as shown in Figure 10. As seen from the figure, the pathways are closely related and are involved in the regulation of the inflammatory response, inflammatory-mediated synthesis, antifibrosis, vascular remodelling, extracellular matrix remodelling, and the reduction of glycogen production. Among them, Akt is an important target. In conclusion, TW may inhibit HIF-1, VEGF, TNF-A and other influencing factors through signalling pathways such as AGE-RAGE, VEGF, HIF-1, relaxation, TNF and insulin resistance, thereby reducing the inflammatory response, antioxidant stress, regulating immune regulation, inhibiting angiopathy, delaying renal fibrosis, repairing podocytes and finally delaying the progression of DN.

## Conclusion

TW has complex chemical components and extensive pharmacological activities. Compared with immunosuppressant, TW has definite efficacy and fewer side effects and adverse reactions. It is widely used in autoimmune diseases and various skin diseases, and is one of the hot natural drugs in the research at home and abroad. This article provides theoretical support for TW to treat DN from the molecular biology level. At the same time, this paper also focuses on the plant itself rather than the extract of TW, aiming to promote further research and provide directions for finding new therapeutic targets.

In summary, a total of 52 active ingredients of TW were screened, with 141 predicted targets, 755 targets for DN, 49 potential targets for TW treatment of DN and 12 key active ingredients. The key compounds are mainly terpenes, of which kaempferol and nobiletin are flavonoids, which highlights the fact that flavonoids cannot be ignored in the study of the efficacy of TW. These compounds affect VEGFA, TP53, PTGS2, TNF, MMP9, Jun, FN1, CXCL8, NOS3, PPARG and other core targets through AGE-RAGE, VEGF, HIF-1, IL-17, relaxin, insulin resistance, TNF and other signalling pathways. Reducing the inflammatory response and antioxidant stress, regulating immunity, improving vascular disease, reducing insulin resistance, delaying renal fibrosis, repairing podocytes, blocking cell apoptosis and other processes jointly improve DN. TW has complex chemical components and extensive pharmacological activities. Compared with immunosuppressants, TW has definite efficacy and fewer side effects and adverse reactions. It is widely used in treating autoimmune diseases and various skin diseases and is a popular natural drug in research. This article provides theoretical support for TW to treat DN at the molecular biology level. At the same time, this paper also focuses on the plant itself rather than the extract of TW, aiming to promote further research and provide directions for finding new therapeutic targets.

## Data Availability

The datasets presented in this study can be found in online repositories. The names of the repository/repositories and accession number(s) can be found in the article/Supplementary Material.

## Abbreviations

TW, *Tripterygium wilfordii*; DN, diabetic, nephropathy; TCMSP, Traditional Chinese Medicine Systems; Pharmacology Database and Analysis Platform; TCM, Traditional Chinese Medicine; OB, oral bioavailability; DL, drug-like; GO, Gene Ontology; KEGG, Genes and Genomes; ROS, reactive oxygen species; ESRD, end-stage renal disease.
